# Putative markers for the detection of early-stage bladder cancer selected by urine metabolomics

**DOI:** 10.1186/s12859-021-04235-z

**Published:** 2021-06-05

**Authors:** Jia-You Lin, Bao-Rong Juo, Yu-Hsuan Yeh, Shu-Hsuan Fu, Yi-Ting Chen, Chien-Lun Chen, Kun-Pin Wu

**Affiliations:** 1 Institute of Biomedical Informatics, National Yang Ming Chiao Tung University, Taipei, 11221 Taiwan; 2 Molecular Medicine Research Center, Chang Gung University, Taoyuan, 33302 Taiwan; 3 Department of Life Sciences and Institute of Genome Sciences, National Yang Ming Chiao Tung University, Taipei, 11221 Taiwan; 4 Department of Biomedical Sciences, Chang Gung University, Taoyuan, 33302 Taiwan; 5 Department of Urology, Chang Gung Memorial Hospital, Taoyuan, 33305 Taiwan; 6 College of Medicine, Chang Gung University, Taoyuan, 33302 Taiwan

**Keywords:** Biomarkers, Metabolomics, Gas chromatography-mass spectrometry, Omics data analysis, Bioinformatics

## Abstract

**Background:**

Early detection of bladder cancer remains challenging because patients with early-stage bladder cancer usually have no incentive to take cytology or cystoscopy tests if they are asymptomatic. Our goal is to find non-invasive marker candidates that may help us gain insight into the metabolism of early-stage bladder cancer and be examined in routine health checks.

**Results:**

We acquired urine samples from 124 patients diagnosed with early-stage bladder cancer or hernia (63 cancer patients and 61 controls). In which 100 samples were included in our marker discovery cohort, and the remaining 24 samples were included in our independent test cohort. We obtained metabolic profiles of 922 compounds of the samples by gas chromatography-mass spectrometry. Based on the metabolic profiles of the marker discovery cohort, we selected marker candidates using Wilcoxon rank-sum test with Bonferroni correction and leave-one-out cross-validation; we further excluded compounds detected in less than 60% of the bladder cancer samples. We finally selected eight putative markers. The abundance of all the eight markers in bladder cancer samples was high but extremely low in hernia samples. Moreover, the up-regulation of these markers might be in association with sugars and polyols metabolism.

**Conclusions:**

In the present study, comparative urine metabolomics selected putative metabolite markers for the detection of early-stage bladder cancer. The suggested relations between early-stage bladder cancer and sugars and polyols metabolism may create opportunities for improving the detection of bladder cancer.

**Supplementary Information:**

The online version contains supplementary material available at 10.1186/s12859-021-04235-z.

## Background

More than 430,000 new cases of bladder cancer are diagnosed worldwide, accounting for approximately 165,000 deaths each year [1]. The earlier this cancer is diagnosed and treated, the better the outcome. But early detection of bladder cancer is still challenging. The current diagnosis of bladder cancer is primarily detected by urine cytology and cystoscopy [2]. Urine cytology is a non-invasive test to identify abnormal cells in urine using a microscope. It is a low-cost approach with high sensitivity for high-grade tumors but low sensitivity for low-grade tumors [3–5]. Cystoscopy is an invasive test that uses an endoscope to check for diseases of the bladder. The sensitivity and specificity of cystoscopy vary depending on the stage and grading of the tumor [6]. Although a combination of urine cytology and cystoscopy provides the best current effectiveness for identifying bladder cancer, they are usually not included in regular health exams, which are the primary way to detect early-stage cancer. Patients with early-stage bladder cancer are often asymptomatic, so they have no incentive to take urine cytology and cystoscopy.

Urinary biomarkers provide a promising alternative for the detection of early-stage bladder cancer. Urine samples can be easily obtained by non-invasive urine tests, which are usually a part of routine health checks. Besides, urine is a valuable resource of bladder cancer detection because it is in direct contact with bladder epithelial cells and therefore contains as many compounds or molecules released by tumor cells as possible. Several proteins were detected in urine and reported as biomarkers to the bladder cancer (FDA-approved or non-FDA approved), such as NMP22, Complement factor H-related protein, BLCA-4, and apolipoproteins [7]. The overall sensitivity and specificity of these protein markers are in between urine cytology and cystoscopy. When we use these markers to detect early-stage bladder cancer, however, their sensitivity significantly decreases. A possible reason for the sensitivity drop is that the concentration of these markers is usually at a low level at the early stage of bladder cancer, which makes them difficult to be detected in urine samples and consequently lower the chance to identify cancer patients. Thus, we need better biomarkers, especially those up-regulated in bladder cancer, to increase the concentration of markers in the urine sample of patients with early-stage bladder cancer.

In addition to genetic or protein markers, metabolites are also useful marker candidates due to their essential role in various pathways. Comparative urine metabolomics has been used to identify metabolite markers for bladder cancer and has found some, such as lactate, phosphocholine, and adenosine [7]. Metabolomics is a systematic study to profile metabolites of biological samples; comparative metabolomics mainly aims at performing differential analysis on different metabolic statuses to capture the changes affected by factors such as environmental, physiological, pharmaceutical, or diseases [8]. Identifying and quantifying metabolites is an essential step in metabolomics. Analytical chemists have developed high-throughput techniques to identify and quantify compounds or molecules in omics research, such as liquid chromatography-mass spectrometry (LC–MS), gas chromatography-mass spectrometry (GC–MS), or nuclear magnetic resonance (NMR) [9]. Among these techniques, GC–MS is suitable for metabolomics due to its high sensitivity, peak resolution, and reproducibility [10]. GC–MS have been used to find metabolite markers for pancreatic cancer [11], lung cancer [12], and influenza A [13], etc. There have been several settings proposed for GC–MS, in which two-dimensional gas chromatography-mass spectrometry (GC × GC–MS) provides the best chromatographic peak capacity, selectivity, and sensitivity for the analysis of small molecules [14]. Among all configurations of GC × GC–MS, two-dimensional gas chromatography coupled to a time-of-flight mass spectrometer (GC × GC–TOFMS) receives the best analytic results [15, 16]. There were studies comparing urine metabolic profiles from patients with bladder cancer and healthy subjects by GC × GC–TOFMS and reporting several metabolite markers such as taurine, citrate, and succinate [17–19]. Considering all the above mentioned, we here performed a GC × GC–TOFMS-based comparative metabolomics analysis on urine samples to find metabolite marker candidates for the detection of early-stage bladder cancer, and hopefully, these putative markers may help us gain insight into the metabolism of early-stage bladder cancer.

The inguinal hernia was frequently seen in the urologic ward. The protruding inguinal lump was surgically corrected to reduce and restore the bowel content with the support of the anatomic structure. It was essentially a procedure of surgical correction of the weakened facial structure. Most of the inguinal hernia patient in the urological ward was in age comparable to bladder cancer patients which provide a good cohort as the control group. In the present study, we recruited 63 patients diagnosed with early-stage bladder cancer as our cases and 61 patients diagnosed with hernia as our controls. All eligible hernia patient was recruited with the criteria of no history and no sign of any cancer. We used GC × GC–TOFMS to analyze the urine samples of these 124 patients and identified 922 compounds. The metabolic profiles of the 922 compounds of 100 patients were subjected to computational procedures to select marker candidates; we further used the metabolic profiles of the remaining 24 patients to evaluate the performance of the selected markers. We selected eight putative markers, one of which cannot be identified as any metabolite and annotated as ‘unknown;’ the others were identified and annotated as the following seven metabolites: desaminotyrosine, erythritol, d-ribose, ribitol, d-fructose, d-mannose, and d-galactose. Intriguingly, the abundance of all the eight markers was high in our bladder cancer samples but extremely low in hernia samples. The up-regulation of these putative markers in cancer samples suggested hypothetical relations between early-stage bladder cancer and sugars and polyols metabolism.

## Results

### Data preprocessing

Our GC–MS analysis identified 922 compounds from our samples (Additional file 1: Table S1). Metabolomic data are sensitive to a variety of factors, such as the changes in the patient’s food intake or lifestyle. So, in our data, there were significant differences among different patients’ metabolic profiles; not all the 922 compounds were detected in every sample, leading to many missing values in our profiles. We dealt with the missing-value problem by assigning zero as the abundance of undetected compounds.

### Different metabolic profiles between bladder cancer and hernia

**Fig. 1:**
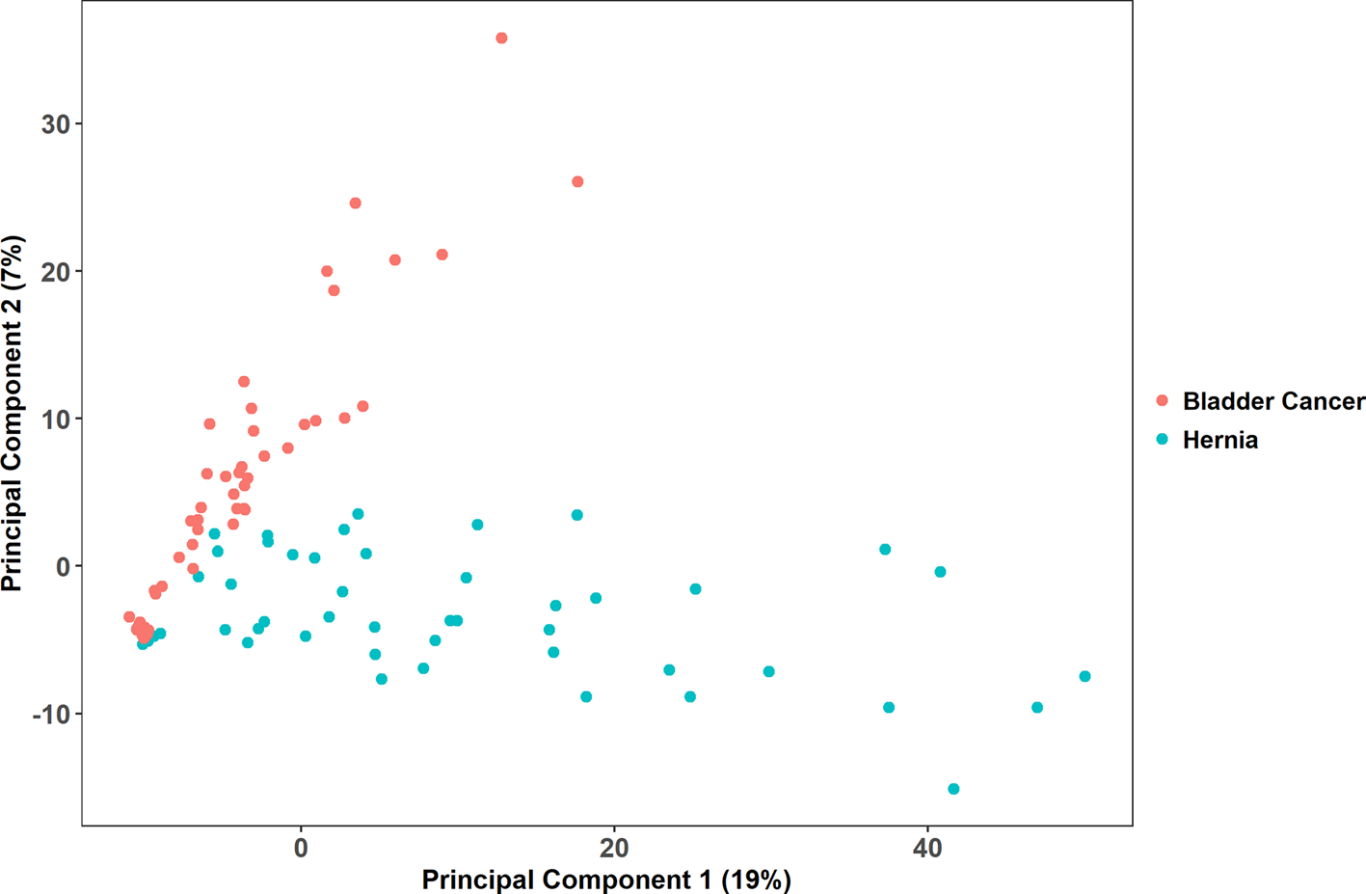
Score plot of PCA. Each point represents a profile of 922 compounds. The value of the first principal component of the PCA goes on the horizontal axis, and the value of the second principal component goes on the vertical axis. This unsupervised analysis captures 0.491 variation between metabolic profiles (R2X = 0.491)

**Fig. 2:**
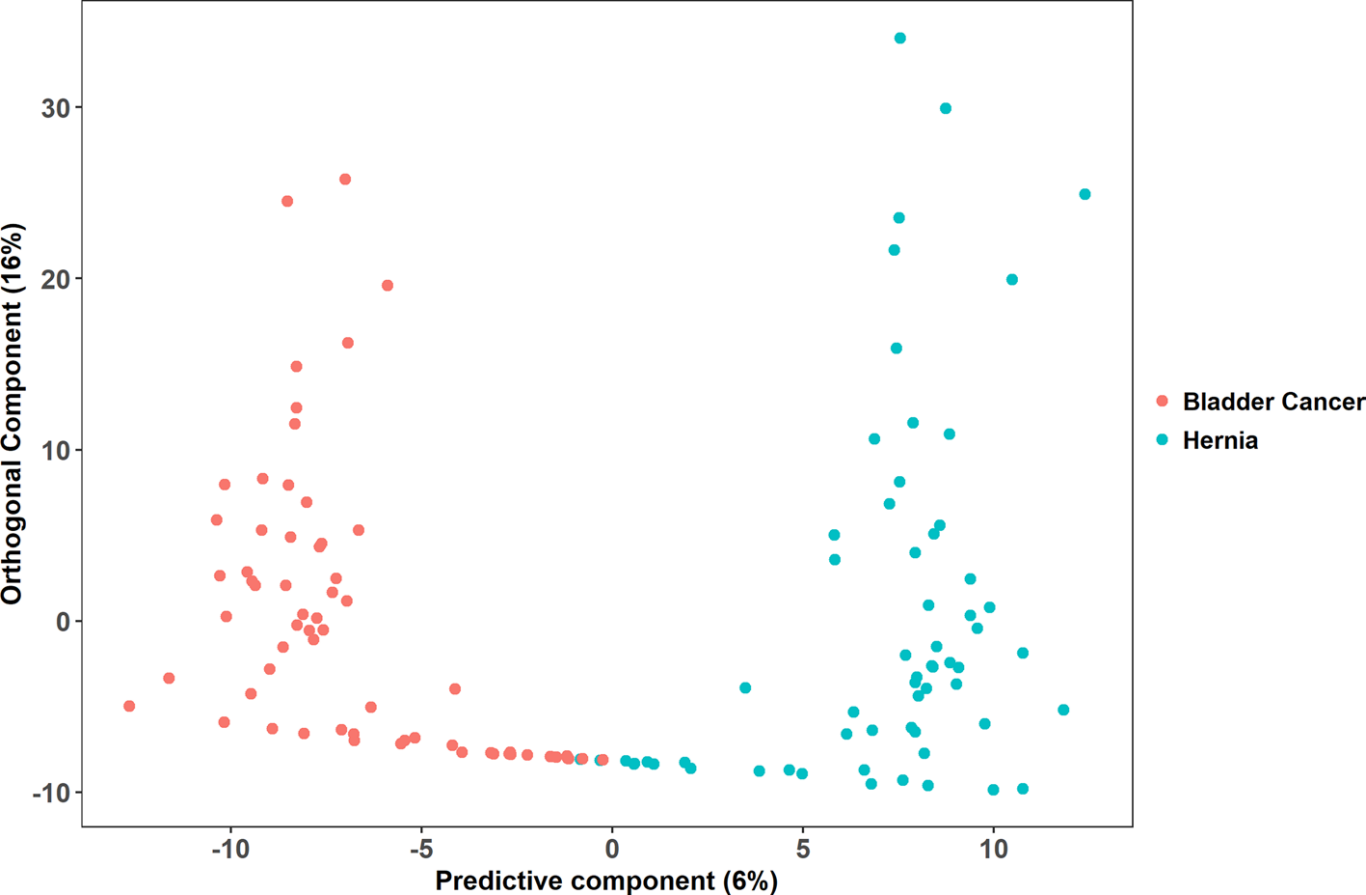
Score plot of OPLS-DA. Each point represents a profile of 922 compounds. The value of the predictive component goes on the horizontal axis, and the value of the orthogonal component goes on the vertical axis. This supervised analysis captures 0.836 variation between metabolic profiles (R2Y = 0.836) and exhibits 0.663 predictive power (Q2 = 0.663)

Based on the profile of the identified 922 compounds, we transformed our samples into high-dimensional data points. We first performed a PCA to examine if the data points were intrinsically distributed into two groups, and its score plot was presented by two principal components (Fig. 1). Though the data points were not perfectly clustered into two separate groups, they showed a bipartite trend in the plot (R2X = 0.491). We followed performing an OPLS-DA analysis to see if our data points could be classified into bladder cancer and hernia, and its score plot was presented by one predictive component and one orthogonal component (Fig. 2). The bladder cancer and hernia points were better separated by OPLS-DA than PCA (R2Y = 0.836, Q2 = 0.663).

### Metabolite marker discovery

**Fig. 3:**
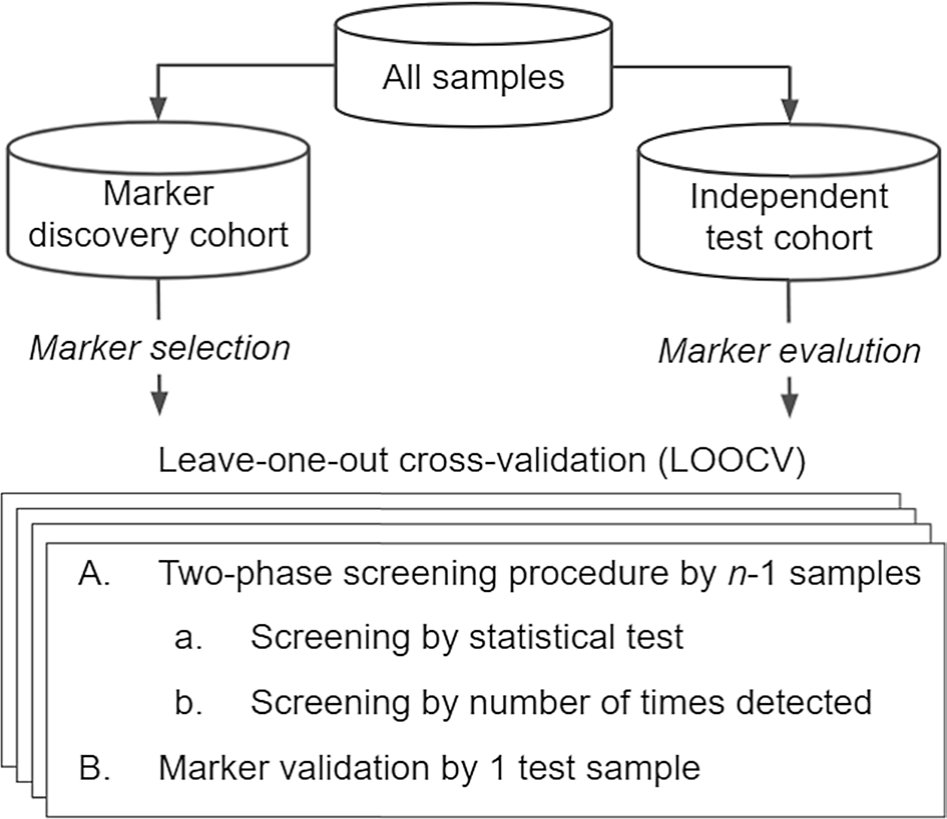
Computational workflow for marker discovery. All samples were partitioned into two cohorts. Marker discovery cohort of size *n* was used for marker selection by a two-phase screening procedure with LOOCV. In LOOCV, (A) markers selection was done using *n*-1 samples and (B) validation using the remaining one test sample. Furthermore, repeating this for *n* times for each sample as the test sample. The independent test cohort was used for independent marker evaluation

The computational workflow of our marker discovery was depicted in Fig. 3. There were three main steps in the workflow. First, we prepared two cohorts; one was for marker selection (100 samples) and the other for independent marker validation (24 samples). Second, we applied LOOCV on the marker discovery cohort to iteratively select discriminating compounds and test their performance. Finally, we used the independent test cohort to perform an independent evaluation to see if the selected compounds could distinguish bladder cancer from hernia samples.

In the first step, all our 124 samples (63 bladder cancer and 61 hernia samples) were randomly divided into two cohorts by sampling probability proportional to size [20]. Marker discovery cohort contained 51 bladder cancer and 49 hernia samples, which was used for marker selection. The independent test cohort contained 12 bladder cancer and 12 hernia samples, which was used for independent marker evaluation. The members of the two cohorts were listed in Additional file 2: Table S2.

In the second step, we applied LOOCV and a marker screening procedure for the marker discovery cohort to select discriminating compounds. Our marker screening procedure was a two-phase pipeline as follows.

1. *Screening by the statistical test* The results of the Shapiro–Wilk normality test told us our data did not fit a normal distribution. We accordingly applied the Wilcoxon rank-sum test with Bonferroni correction to choose compounds whose mean ranks of abundance had significant differences in bladder cancer and hernia groups. We accommodated a rigorous confidence level, p < 5.42e − 5, to make sure that we selected an appropriate number of candidates for further screening.
2. *Screening by the number of times detected* A compound cannot be a useful marker if it cannot be detected in most samples [19]. In the current study, only the compounds that had a non-zero abundance in more than 60% of bladder cancer samples in the marker discovery cohort were chosen as marker candidates.

**Fig. 4:**
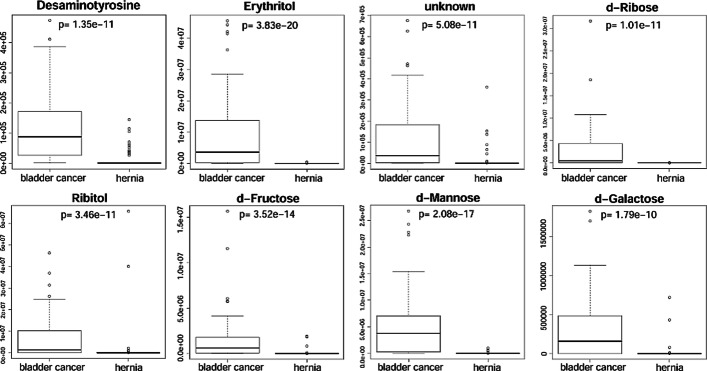
Abundance distribution of putative markers in different groups. The *p* values were reported by the Wilcoxon rank-sum test performed on the marker discovery cohort

**Fig. 5:**
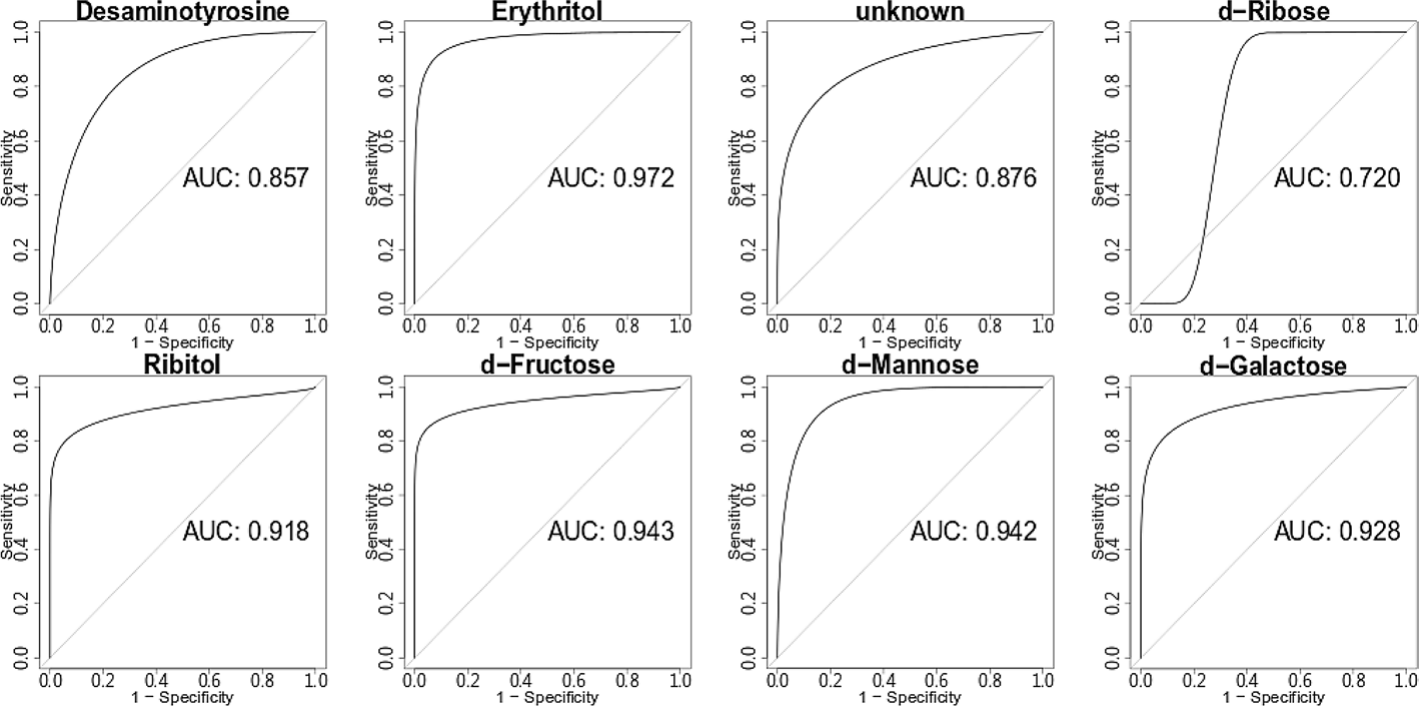
ROC curves of the eight putative markers. All the ROCs and their AUCs were obtained from the marker discovery cohort

Since our screening pipeline was subjected to LOOCV, we obtained 100 lists of candidate markers and 100 prediction results of these lists. Please refer to the Additional file 3: Table S3 for the 100 complete listings of candidate markers and their corresponding prediction results. Each list contained 15 or 16 compounds; we selected compounds that appeared to all the 100 lists and removed those that originated from background contamination of GC–MS. We finally obtained eight putative markers, one of which cannot be identified as any metabolite and therefore annotated as ‘unknown.’ The attributes of the eight markers, along with their prediction accuracy, sensitivity, and specificity reported by the LOOCV were listed in Table 1. Please note that to best predict the test samples, a marker might apply different classifying thresholds in the different rounds of the LOOCV. The abundance distribution of the eight markers in bladder cancer and hernia groups were shown in Fig. 4. The small *p* values reported by the Wilcoxon rank-sum test revealed that the eight markers were differential in the two groups; the eight markers were nearly not detected in the hernia samples of the marker discovery cohort. We also depicted the ROCs of the eight markers in Fig. 5. The AUCs of the eight markers (> 0.850 except d-Ribose = 0.720) showed their discriminating power to distinguish bladder cancer samples from hernia ones. We further determined the classifying threshold of each candidate based on the best combination of the sensitivity and specificity reported by the ROCs.

**Table 1:** Selected putative markers

Compound name	Mass	Metabolite	Accuracy	Sensitivity	Specificity
Benzenepropanoic acid, 4-dihydroxy-, methyl ester(CAS)	107	Desaminotyrosine	0.68	0.61	0.76
3,8,Dioxa2,9,disiladecane,2,2,9,9,tetramethyl,5,6,bis-trimethylsilyl-oxy,R,S,CAS,3	217	Erythritol	0.94	0.92	0.96
Pentitol-1,1-D2,2-desoxy-tetrakis-O(trimethylsilyl)	231	unknown	0.81	0.75	0.88
d-Ribose,2,3,4,5,tetrakis-O-trimethylsilyl-o-methyloxime,CAS	103	d-Ribose	0.74	0.55	0.94
Ribitol,1,2,3,4,5,pentatms,7	217	Ribitol	0.84	0.75	0.94
d,Fructose,1,3,4,5,6,pentakis-O-trimethylsilyl-O-methyloxime-CAS,3	217	d-Fructose	0.84	0.75	0.94
d-Mannose,2,3,4,5,6,pentakis-O-trimethylsilyl-o-methyloxyme-1Z,3	160	d-Mannose	0.86	0.80	0.92
d-Galactose,2,3,4,5,6,pentakis-O-trimethylsilyl-o-methyloxyme,1Z,5	103	d-Galactose	0.80	0.65	0.96

Performance was reported by LOOCV on the marker discovery cohort. The third compound cannot be identified as any metabolite and accordingly annotated as ‘unknown’

In the third step, we evaluated whether or not the eight putative markers were useful for unseen samples by the independent test cohort. We first used the classifying thresholds determined in the marker discovery step to discriminate between bladder cancer and hernia. The test results were listed in Table 2. Overall, the eight markers obtained better specificities than sensitivities in this independent test; all the discriminating specificities were above 0.7, but some sensitivities were below 0.7.

**Table 2:** Discrimination performance of putative markers

Metabolite	Accuracy	Sensitivity	Specificity	Classifying threshold
Desaminotyrosine	0.88	0.83	0.92	6633.20
Erythritol	0.83	0.83	0.83	6713.05
unknown	0.75	0.67	0.83	5616.04
d-Ribose	0.83	0.67	1.00	65,947.18
Ribitol	0.71	0.67	0.75	35,510.49
d-Fructose	0.83	0.83	0.83	37,952.03
d-Mannose	0.96	0.92	1.00	62,727.26
d-Galactose	0.79	0.67	0.92	13,823.33

These markers were evaluated using the independent test cohort. The classifying thresholds, however, were determined based on the marker discovery cohort

**Fig. 6:**
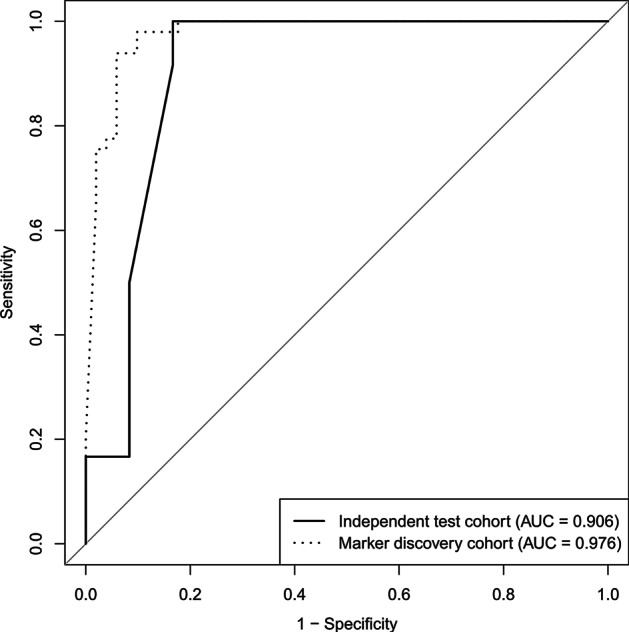
ROC curves of logistic regression. The dotted line represented the ROC curve of the logistic regression model evaluated by the marker discovery cohort and obtained an AUC of 0.976. The solid line represented the ROC curve of the logistic regression model evaluated by the independent test cohort and obtained an AUC of 0.906

To see if we could better discriminate bladder cancer from hernia by combining the eight putative markers, we built logistic regression models using the eight markers. The ROCs in Fig. 6 revealed the performance of the regression models on the marker discovery cohort and independent test cohort. The AUC of the regression model on the marker discovery cohort is 0.976, which reveals that our logistic regression successfully learned from the training data how to discriminate between early-stage bladder cancer and hernia; this model almost perfectly discriminated between the two groups in our training cohort. The AUC of the regression model on the independent test cohort is 0.906. The minor AUC loss, from 0.976 to 0.906, revealed that the learning procedure of our logistic regression model did not overfit, and the model performed well on unseen data.

## Discussion

Among the eight putative markers, ribitol and erythritol are two metabolites of particular interest to us. Both are sugar alcohols whose content increases in urine and plasma of patients with inborn enzymatic deficiency of transaldolase (TALDO) [21]. Patients with TALDO deficiency have several clinical phenotypes, such as liver dysfunction, hepatosplenomegaly, dysmorphism, anemia, and thrombocytopenia [22]. TALDO is the rate-limiting enzyme of the non-oxidative reactions of the pentose phosphate pathway (PPP). There are two primary functions of the non-oxidative reactions of the PPP; one is to synthesize ribose-5-phosphate required for nucleotide synthesis, the other is to recycle and convert pentose-5-phosphate into intermediates of glycolysis. Coincidentally, ribitol and erythritol also have extraordinarily high levels in patients’ urine with transketolase (TKT) mutation and ribose-5-phosphate isomerase (RPI) deficiency [23, 24]. Both TKT and RPI are also enzymes involved in the non-oxidative steps of the PPP. Thus, we conjecture that the development of early-stage bladder cancer might be in correlation with the mutation and downregulation of enzymes of non-oxidative PPP in bladder cells.

Similar to our conjecture, Uribarri et al. detected the downregulation of TALDO in lung cancer tissues [25], and Hanczko et al. found that mice with TALDO deficiency are more susceptible to acetaminophen-induced liver failure, cirrhosis, and hepatocellular carcinoma (HCC) [26]. Besides, early-onset HCC was also reported to be a novel phenotype of patients with homozygous TALDO1 mutation [27]. The observations possibly can be explained by that the disrupted recycling of the PPP products would lead to a decrease in the reduced form of nicotinamide-adenine dinucleotide phosphate (NADPH) and, in turn, causes the oxidative damage-induced pro-tumorigenic inflammation in the liver [28].

The overexpression of aldose reductase (AR) is another possible reason for the high-level of ribitol. AR was proposed to link with PPP because it converts accumulated C5 sugars to polyols under the absence of TALDO [29]; this process would further deplete NADPH and glutathione. AR is frequently overexpressed in several types of tumor tissues and closely associated with oxidative stress and inflammatory carcinogenesis [30]. Inhibition of AR was shown to suppress tumor cell proliferation, invasion, angiogenesis, and the NF-κB/AP1-dependent inflammatory signaling pathway [30]. Therefore, dysregulation of AR is another possible factor inducing the development of early-stage bladder cancer.

The other four putative markers, d-ribose, d-fructose, d-mannose, and d-galactose are pentoses and hexoses rarely found in the urine of healthy people. The high abundance of the four metabolites in the urine of the patients with bladder cancer may suggest a correlation between sugar metabolism and carcinogenesis of bladder cancer. Though a high level of sugars in the blood may not directly imply diabetes mellitus, some studies revealed the relationship between diabetes mellitus and risk of bladder cancer [31–33]. Hyperglycemia and hypergalactosemia would trigger the AR-mediated oxidative stress and inflammation, and under this stress condition, more than 30% of glucose would be reduced and enter the polyol pathway [34]. The accumulation of polyols in cardiomyocytes was reported to induce expression and activation of AR and activate the JNK/c-jun signaling pathway that is frequently associated with tumor progression [35]. In summary, most of our putative markers seem closely related to the interplay between sugars and polyols metabolism.

As for the remaining two putative markers, one cannot be identified and the other is desaminotyrosine. The literature report about desaminotyrosine is scarce only suggesting a metabolite of the gut microbes related to the immune system [36]. The findings shown in our study suggesting desaminotyrosine as a potential biomarker of bladder cancer await further validation.

Notably, half of our bladder cancer patients have hematuria (30 out of 63), and none of our hernia patients have hematuria. Hematuria is the most common presenting symptom of bladder cancer, especially painless gross (visible) hematuria. The incidence of bladder cancer among those with gross hematuria is 10% to 20%, while only approximately 2% to 5% among patients with microscopic hematuria. However, hematuria may be caused by many disease entities such as urinary tract infections. In this sense, hematuria may be a cancer-related sign instead of a confounding factor. Based on our findings, the 8 putative biomarkers could differentiate hematuria (ROC: 0.781, data not shown), which implied that these markers contain information to identify a common symptom related to early-stage bladder cancer to a certain degree. Moreover, as shown in Fig. 6, the 8 markers differentiated early-stage bladder cancer in a ROC of 0.976, which means that the 8 markers contain extra cancer-specific information to not only identify cancer samples with hematuria but also those without hematuria.

## Conclusions

In summary, we identified 922 compounds and further selected eight putative markers using comparative urine metabolomics. The eight markers also suggested relations between early-stage bladder cancer and sugars and polyols metabolism. The result of this study may create opportunities for improving the detection of bladder cancer.

Though the eight putative markers perform well in our validation, their discriminating power requires further validation since our validation cohort is relatively small. Even if these putative markers exhibit discriminating power from the computational point of view, we need carefully examine their true endogenous origins of substances to see whether they are qualified markers. An important example is the disqualification of the putative in vivo origin of isoprene, which is repeatedly reported as a metabolic marker of cancer, cardio-pulmonary diseases, and rare genetic disorders in human [37].

Please note that there has never been a biomarker so powerful to diagnose a disease; disease diagnosis was made base on clinical presentation and evidence to support and confirm the diagnosis. But a strong correlation of a biomarker and a disease makes the diagnostic “potential” of this marker possible. In this study, we presented data working on a very important clinical scenario and the clues of these possible markers. The eight putative markers will be subjected to further investigation to see if they can be used for diagnosis or used to perform non-invasive monitoring of metabolic effects under certain pathological conditions and any response to administered therapy.

## Methods

### Urine samples from patients with bladder cancer or hernia

All urine samples were collected at Chang Gung Memorial Hospital, Taoyuan, Taiwan. The study protocol was approved and conducted in accordance with the guidelines and regulations of the Medical Ethics and Human Clinical Trial Committee at Chang Gung Memorial Hospital (IRB approval number 107-0960D). Informed consents were obtained from all individual participants involved in the study. The methodology of urine collection has been previously reported [38]. Briefly, first morning urine samples were routinely collected from bladder cancer patients and control patients without a history of urological disease. Urine samples were collected in the presence of a protease inhibitor cocktail tablet (one tablet/50 mL of urine; Roche, Mannheim, Germany) and sodium azide (1 mM). The collected samples were centrifuged at 5000 × g for 30 min at 4 °C within 5 h to remove cells and debris, and the clarified supernatants were stored at -20 °C for further processing.

Urothelial carcinoma is the most common cancer of the urinary bladder. According to the American Joint Committee on Cancer (AJCC) staging system, 8th edition, bladder cancers in stage Ta or T1 are non-muscle-invasive diseases [39]. In this study, we recruited 63 early-stage bladder cancer patients diagnosed with non-muscle-invasive diseases. The diagnosis of bladder cancer was all pathologically proven of urothelial carcinoma after transurethral biopsy or resection of the tumor. The urine was discarded and excluded for further analysis if the diagnosis was not confirmed. All the bladder cancer samples were from primary tumors. We also recruited 61 patients diagnosed with hernia and without past cancer histories as our control cohort. All the 124 patients are of comparable age and underwent exactly the same procedures of urine sample collection on the first morning after admission before surgical intervention. Patient demographics of this study were summarized in Table 3. We compared numerical and categorical variables using the Wilcoxon signed-rank test and chi-square test, respectively; the statistical analyses were conducted by SAS 9.4. To eliminate possible bias from batch effect, the sample was processed and analyzed randomly. The metabolomic analysis was performed in triplicate, and the operator was not aware of the status of the samples.

**Table 3:** Patient demographics

	Bladder cancer	Hernia	***p*** value
Number of subjects	63	61	
Age	67.4 ± 13.5	65.0 ± 12.0	0.1834
*Gender*			0.0005
Male	45	58	
Female	18	3	
*Risk factor*			
Hypertension	35	23	0.0504
Diabetes Mellitus	11	5	0.1802
Smoke	19	11	0.1434
Hematuria	30	0	< 0.001

*P* values were reported by the Wilcoxon signed-rank test and chi-square test for numerical and categorical variables, respectively

### Chemicals

Protease inhibitor cocktail tablet was purchased from Roche (Mannheim, Germany). Sodium azide, urease, methoxyamine, pyridine, N,O-Bis(trimethylsilyl)trifluoroacetamide with 1% trimethylchlorosilane (BSTFA + 1% TMCS), and methanol were purchased from Sigma-Aldrich (St. Louis, MO). Methoxyamine was prepared in pyridine at a concentration of 15 mg/ml.

### Urine sample derivatization

We used the protocol common to urine metabolomics research to perform urine sample derivation [40, 41]. The urine samples were collected in the presence of a protease inhibitor cocktail tablet (one tablet per 50 mL urine) and sodium azide (1 mM in urine). Cells and debris were removed by centrifugation (5000 g for 30 min at 4 °C). This was done within one hour after the sample collection. The sample was then kept at −80 °C for long-term storage. Before GC–MS analysis, the sample was thawed at 4 °C. Freezing-point depression was measured to determine the osmolality of samples using the model 3320 osmometer of the Advanced Instruments (Norwood, MA). All the samples were normalized by diluting their osmolalities to 250 mOsm/kg. 100 μL of urine was centrifuged at 12,000 g, 5 °C for 10 min, 50 μL of supernatant was added with 30 units of urease and incubated at 37 °C for 15 min to decompose and remove excess urea present in it. 170 μl of methanol was then added to it. The solution was vigorously vortexed for 30 s and was centrifuged at 12,000 g, 5 °C for 5 min. 200 μl of supernatant was transferred to a GC vial and evaporated to dryness under nitrogen at room temperature. 80 μL of methoxyamine in pyridine (15 mg/ml) was added to each GC vial. The solution was then vigorously vortexed for 30 s. Methoximation reaction was performed at 30 °C for 30 min. Silylation was performed by adding 80 μL of BSTFA (1% TMCS) under 70 °C for 1 h. The sample was used for GC–MS analysis.

### GC–MS analysis

We applied the procedure used by Hua et al. to perform GC–MS analysis [42]. GC–MS was performed using the Pegasus® 4D GC × GC-TOFMS from LECO (St. Joseph, MI). The separation conditions on an Rtx-5MS column (30 m × 0.25 mm × 0.25 μm) for GC–MS analysis was set as follows: column flow, 1 mL/min helium; injector temperature, 250 °C; GC temperature program: 40 °C for 1 min, increase to 300 °C in 10 °C/min changes, 300 °C for 8 min; solvent delay, 440 s; transfer line temperature, 300 °C; pegasus acquisition rate, 10 spectra/sec; mass range saved: m/z 50–800; ion source temperature: 200 °C.

The data were analyzed using the program ChromaTOF® Software from LECO (St. Joseph, MI), by setting the threshold for up to 650 hits on m/z similarity and compared with the metabolites present in libraries of the software. Compounds with peak-areas below 700 were undetectable and compounds with less than 650 hits for m/z identification were removed. The annotated synthetic compounds were removed.

### Computational and statistical tools

#### Test of clustering

The visualization of some dimensionality reduction or regression algorithms, such as principal components analysis (PCA), partial least squares projection to latent structures (PLS), partial least squares discriminant analysis (PLS-DA) and orthogonal partial least squares discriminant analysis (OPLS-DA), has been used to intuitively inspect the possible separation of metabolomics data [43]. In the current study, we were also wondering if our bladder cancer samples could be discriminated from hernia samples based on metabolic profiles. We subjected our metabolic profiles to both PCA and OPLS-DA analysis. PCA is an unsupervised multivariate analysis that we used to verify if metabolic profiles were intrinsically distributed into two groups. The PCA reported the R2X value that represented the explained variation between metabolic profiles. On the other hand, OPLS-DA is a supervised multivariate analysis that we used to capture the separation of our bladder cancer and hernia samples. The OPLS-DA reported the R2Y and Q2 values. R2Y represented the explained variation between metabolic profiles; Q2 represented the predictive power of the model. The PCA and OPLS-DA analyses were carried out by R 4.0.0 with the package ropls 1.20.0, and its visualization was produced by the package ggplot2 3.3.1.

#### Statistical test

We performed the Shapiro–Wilk normality test [44] to make sure if our data are normally distributed. The result of the normality test showed that our datasets were not characterized by any parameter (*p* < 1.17e-7). We therefore used the Wilcoxon rank-sum test [45] to verify if the median ranks of bladder cancer and hernia groups were statistically different. All the tests in this study were carried out with Bonferroni correction [46] to avoid type I errors, i.e., false positives, to a maximum extent; we only wish to identify a few markers with high statistical significance. All the statistical tests were carried out by R 4.0.0 with the package stats 4.0.0.

#### Prediction model

Since the discrimination between bladder cancer and hernia is a dichotomous problem, we used logistic regression [47] to build prediction models for the detection of early-stage bladder cancer. We used the generalized linear function with logit link function to implement our logistic regression in R 4.0.0.

#### Performance measures

We used accuracy, sensitivity, specificity, and the area under the receiver operating characteristic curve (AUC) as our performance measures. Accuracy is the probability that a sample is correctly predicted; sensitivity is the probability that a positive sample (bladder cancer in the present study) is correctly predicted; specificity is the probability that a negative sample (hernia in the present study) is correctly predicted. The receiver operating characteristic (ROC) curve plots sensitivity versus (1 − specificity) at different classifying thresholds. The AUC represents the area beneath the ROC curve, which is widely used to evaluate the performance of a binary classifier. The AUC ranges from 0 to 1; 0 represents 100% wrong prediction, 1 represents 100% correct prediction, and 0.5 represents random guess. Our AUC analysis was carried out by R 4.0.0 with the package pROC 1.16.2.

We used leave-one-out cross-validation (LOOCV) to assess the stability of our marker performance. In LOOCV, we used one sample for marker evaluation and the remaining samples for marker selection. The procedure will repeat *n* times if there are *n* samples, to exam all possible *n* combinations of one sample and *n* – 1 samples. The LOOCV was carried out by R 4.0.0.

## Supplementary Information

**Additional file 1: Table S1.** The 922 compounds identified from 124 patients with bladder cancer or hernia.

**Additional file 2: Table S2.** The members of marker discovery cohort and independent test cohort.

**Additional file 3: Table S3.** Putative markers and their corresponding prediction results.

## Data Availability

The data sets supporting this study are included in this article and its supplementary files.

## Abbreviations

GC–MS: Gas chromatography-mass spectrometry
GC × GC-TOFMS: Two-dimensional gas chromatography coupled to a time-of-flight mass spectrometer
TALDO: Transaldolase
AR: Aldose reductase
OPLS-DA: Orthogonal partial least squares discriminant analysis
LOOCV: Leave-one-out cross-validation
ROC: Receiver operating characteristic
AUC: Area under the receiver operating characteristic curve
